# BirdsEyeView (BEV): graphical overviews of experimental data

**DOI:** 10.1186/1471-2105-13-S15-S11

**Published:** 2012-09-11

**Authors:** Lifeng Zhang, Daniel Berleant, Yi Wang, Ling Li, Diane Cook, Eve Syrkin Wurtele

**Affiliations:** 1 Department of Electrical and Computer Engineering, Iowa State University, Ames, Iowa, 50011, USA; 2 Department of Information Science, University of Arkansas at Little Rock, Little Rock, AR, 72204, USA; 3 Department of Statistics, Iowa State University, Ames, Iowa, 50011, USA; 4 Department of Genetics, Cell & Development Biology, Iowa State University, Ames, Iowa, 50011, USA

## Abstract

**Background:**

Analyzing global experimental data can be tedious and time-consuming. Thus, helping biologists see results as quickly and easily as possible can facilitate biological research, and is the purpose of the software we describe.

**Results:**

We present BirdsEyeView, a software system for visualizing experimental transcriptomic data using different views that users can switch among and compare. BirdsEyeView graphically maps data to three views: Cellular Map (currently a plant cell), Pathway Tree with dynamic mapping, and Gene Ontology http://www.geneontology.org Biological Processes and Molecular Functions. By displaying color-coded values for transcript levels across different views, BirdsEyeView can assist users in developing hypotheses about their experiment results.

**Conclusions:**

BirdsEyeView is a software system available as a Java Webstart package for visualizing transcriptomic data in the context of different biological views to assist biologists in investigating experimental results. BirdsEyeView can be obtained from http://metnetdb.org/MetNet_BirdsEyeView.htm.

## Introduction

Rapid technological innovation is enabling new biological approaches, and accelerating biologists' ability to collect large scale transcriptomics, metabolomics and proteomics data. The important trend emphasizing deposition of data and metadata in publicly-available databases means that this omics data is available to all (e.g. [1-4]). Extraction of data and hypotheses about that data has become key to biological research. For example, analysis of global and massive datasets has led to a number of discoveries on gene function (e.g. [5]).

Because the time it takes to investigate massive data manually and extract useful biological knowledge is often prohibitive, functional genomics tools for data analysis and visualization are critical and many approaches have been or are being developed. Some approaches apply only to single experiments or limited data, whereas others can handle multiple experiments and large biological networks. Histograms and line graphs have been used to show experimental values for many samples at once, e.g. Caryoscope [6-8]. Heat maps have been integrated with dendrograms to display microarray data and cluster it using different algorithms, as in Hierarchical Clustering Explorer [9-15].

Data analysis can provide powerful views of raw data. However, it reveals less about the overall biological context, such as pathway and cellular information at various granularities. Another approach to visualizing experimental data is to integrate it into a biological network graph. Reactome [16] shows a single metabolic pathway at a time, using node color to represent expression. Ingenuity Pathway Analysis [17] uses green and red colors to indicate up- and down-regulated genes respectively in pathway network graphs. Omics Viewer [18] displays omics data in which biomolecules in metabolic pathways are color-coded based on expression level. MapMan [19] shows metabolic pathways by using groups of tiny colorful squares on different locations of the pathway network graph to represent groups of biomolecules participating in different places. CytoScape [20] includes powerful graph analysis, and a variety of network layout algorithms, and relies on user-input annotations, pathway or network data, and experimental data. GENeVis [21] uses a tiny group of bars to show the expression data group in regulatory and metabolic pathway network nodes. VistaClara [22] shows bars grouped beside each biological network node, and GenMAPP [23] uses a strip for each node (box) in the pathway to group different expression levels at different times for that node. However, even tiny bars or groups of squares are large relative to nodes themselves, hence the graph size needed for a whole network to be visualized can become quite large. MetNetGE [24] uses Google Earth-based software to divide networks of metabolic pathways into layers to represent information such as cellular location and to color biomolecules in the network graph according to user-input experimental data. This tool can obtain pathway information from MetaCyc [25] or from MetNetDB [26]. An interesting combined view of experimental data overlays of a single pathway in the context of a user-provided network with cellular layers can be viewed using Cerebral, a plug-in for Cytopscape [27]. BiGGEsTS [28] uses a tree-like graph to display a hierarchy of Gene Ontology terms, in which colors represent the expression value. However, the user cannot access detailed information about each gene.

Here we introduce a software application to help biologists analyze transcriptomics including RNA-Seq and microarray data. This tool, BirdsEyeView (BEV) links a graphical Cellular Map view, a Pathway Tree view, and a Gene Ontology view together to display experimental results. In addition to incorporating statistical analysis, BEV focuses on the interactive display of experimental entities, in this case transcripts, in different locations of the Cellular Map, in a dynamic Pathway Tree, and using Gene Ontology views based on the each entity's annotation information. As such, it gives biologists a graphical "Birds Eye View" of experimental data, hence its name, "BirdsEyeView."

BEV is a part of the MetNet platform, and is designed to visualize biological information and place that information within a biological context. The MetNet (Metabolic Network Exchange, metnetdb.org) bioinformatics platform containing BEV is a suite of software applications designed for analysis of genomic, proteomic, transcriptomic, and metabolomic experimental data [26,1,29]. MetNet applications use statistical and graphing techniques as well as innovative visualization to help users analyse biological networks.

At the center of the MetNet platform is the MetNet database, MetNetDB. MetNetDB includes an integrated metabolic and regulatory network for Arabidopsis based on extant databases and human curation. Network and pathway data for other photosynthetic organisms, (e.g. soybean, grape, and medicinal species), humans, and yeast are in development. The MetNetDB structure integrates biochemical interactions, metabolic pathways, regulatory networks, genome information, Gene Ontology and other relevant annotation, to enable formulation of testable hypotheses about transcriptomic, proteomic, and metabolomic data [26]. The MetNet database is updated daily from the source databases.

**Figure 1:**
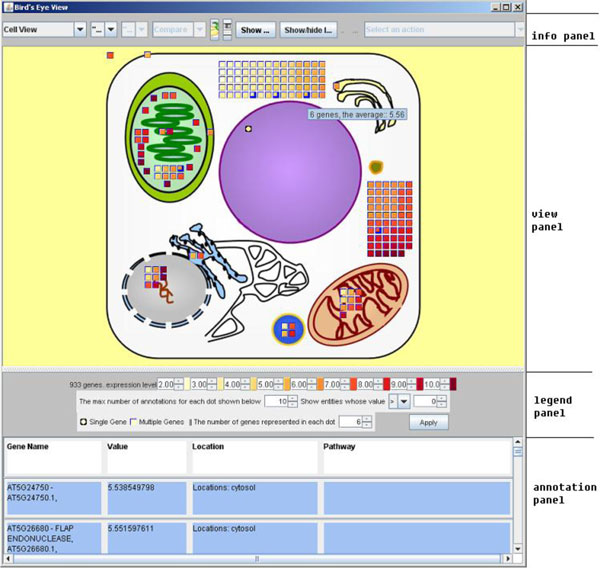
**Main interface of BirdsEyeView**. From top to bottom: information panel, view panel, legend panel, and annotation panel.

BEV directly accesses the information stored in MetNetDB, together with transcriptomics data in a user-uploaded file, to provide users with overviews of their data in the context of pathways, subcellular location and Gene Ontology terms. It provides unique views of experimental results by mapping transcripts to different views based on those entities' annotation in MetNetDB. In addition, it graphically (using color, distribution of entities among compartments, and a size) indicates experimental differences among entities across different compartments in different views. Currently, BEV provides three views: a Cellular Map on a fixed cellular diagram, a Pathway Tree in which views are dynamically displayed as nested rectangles, with different sizes representing different pathways, and Gene Ontology Hierarchy which provides a series of hierarchical rectangles mapped to Gene Ontology classifications (Figure 1). BEV maps the input data to these views to help users develop hypotheses about their data. The "Birds Eye View" enables users to interactively understand the distribution of experimental results across different granularities, cellular compartments, pathways, and Gene Ontology levels. Users can quickly view significantly overrepresented or underrepresented transcripts in this context. Details about each entity can be retrieved from the graphical view.

A user's choice of visualization and analysis software for transcriptomics data will depend on the expertise and biological interests of that user. BEV incorporates basic statistical analysis, and also allows the user to conduct an appropriate statistical analysis using other software and visualize the results of this analysis graphically. This provides a biological context based mostly on experimentally-based cellular location and pathway information while giving users links to the network information in MetNetDB.

Distinctions between BEV and existing software include: 1) Dynamic chunking enables experimental data with detailed annotation to be displayed without occupying huge spaces; 2) BEV provides an interactive overview of the distribution of a whole set of experimental transcriptomic data in the context of cellular, pathway, and Gene Ontology views; and 3) BEV enables a visual comparison of experimental results in which the user can select which combined view of memberships to visualize. As a result, BEV is a quick and easy tool for aiding biologists in hypothesis formulation, and for providing a first look at the data in a biological context.

## Methods and implementation

### Program overview

BEV is a stand-alone Java application that accepts user input data and gives users interactive "Birds Eye Views" of their input data by mapping the data to various views. For demonstration purposes, BEV makes it easy to load a default sample dataset from the MetNet Website, so that the program does not require user-generated data files to try the software. This test dataset is from experiments on perturbations of RNA accumulation associated with stress, provided by Mittler [30].

BEV reads a list of biological entity IDs in the input file and finds the corresponding biological annotations by queries to the MetNetDB database including gene name, location, pathway, and Gene Ontology information. The information in MetNetDB is regularly updated from different databases and analyses. Based on the annotation information, the program displays the RNAs in the input file as small graphical icons (circles or rectangles) in different places in the different views. The program adjusts icon colors based on the average expression value of the entity represented by that icon. As such, patterns of experimental input will be shown in different views so that users can view the distribution of highly accumulated RNAs across compartments, pathways, and Gene Ontology Processes. Users can switch and combine views and experiments to see different views of the data. The user can view individual samples, or compare data from any two samples in the input data file.

### Input data

BEV can display experimental results, such as transcriptomic analyses. For either RNA-Seq or microarray analysis, data can be input as a single, combined file or as separate files. For RNA-Seq data, the input files are a file mapping values to gene IDs/locus IDs, a file containing a list of sample, and a file of numbers describing the values of experimental results. For microarray results, files are a chip list file containing a list of Affymetrix probe IDs (chip probes) in the experiments, a file holding the list of samples, and a file of numerical values of experimental results.

If the user processes data by subjecting it to statistical screening before submitting as input, BEV could be used to analyse a data set containing only genes whose data meets a desired quality threshold. For example, statistical analysis could be applied to identify genes that are differentially expressed relative to a control, and only genes with *p*-values below a certain threshold retained.

## Results

The main interface is divided into four panels (Figure 1): an information panel, a view panel, a legend panel, and an annotation panel. Users upload data files via the information panel. Upon loading data, BEV maps the data to icons in the views of the main interface. Within the information panel, users can operate buttons and drop-down lists to switch between samples and views, and perform other view-related operations.

The view panel is the largest, and holds different views (displayed one at a time by default, or in combination when the viewer selects "Show two views together"). The proteins encoded by the transcripts specified in the user's input file are represented as icons inside each view.

The legend panel contains a legend and user options for customizing view panel. User options include changing color range values, and user-selected filters for biomolecular entities whose data values are over or under the selected values. In addition, users can select the maximum number of entities to be represented per icon, and the maximum number of entities to show in the annotation panel before opening a new frame. The number of entities per icon has a view scope based minimum value based on the number of entities in each cell compartment and the sizes of the compartments, such that all compartments can display all entities that they contain.

The annotation panel at the bottom of the screen shows detailed information about entities selected by the user from the view panel icons.

### Cellular map view

**Figure 2:**
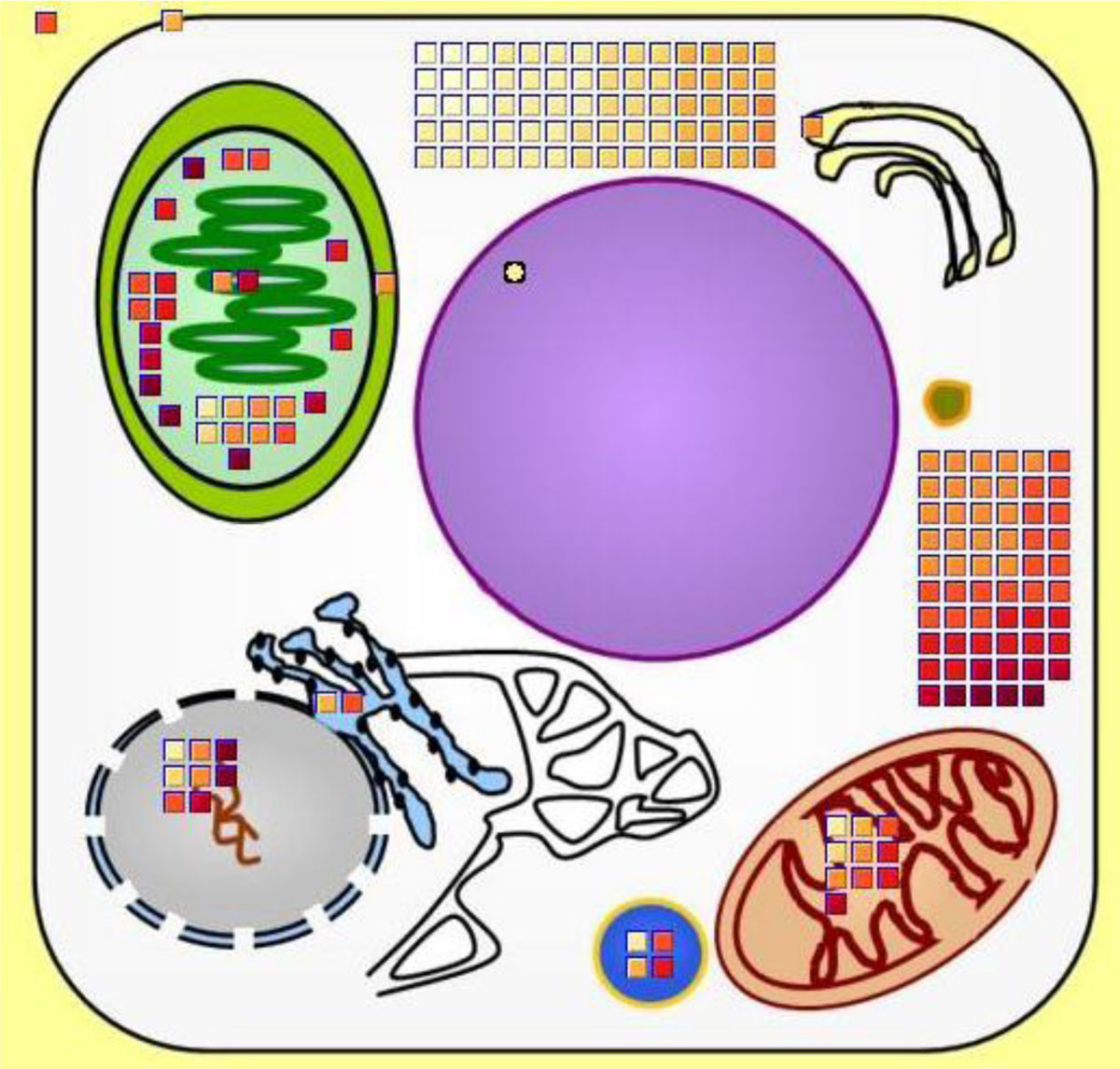
**Cellular map view of experimental data within BirdsEyeView**.

In this view, an image showing the structure of a plant cell is displayed. See Figure 2. Major cell components are shown including nucleus, vacuole, mitochondria/cristae, golgi apparatus, endoplasmic reticulum, apoplast, plastids/thylakoids, chloroplast, cytosol, and plasma membrane.

After loading the input files, the program displays entities from the list of input files in appropriate compartments with graphical icons. Entity icons are placed in compartments based on the location information about each entity loaded from MetNetDB. The locations represented in the cellular map view indicate the known or suspected location within the cell of the protein corresponding to the transcript. Entities whose location information is not known are not displayed. If there are possible multiple locations for an entity, then the entity will be shown in multiple compartments.

### Color coding the icons

Because an important function of BEV is to display the user's transcriptomics data in a biological context, the icons are color-coded according to the corresponding values in the transcriptome data. The program uses the YIOrRd (light yellow to orange to dark red) color system of the RColorbrewer color combination strategy (http://casoilresource.lawr.ucdavis.edu/drupal/node/192, [31]) to represent different data values (e.g. expression levels, log ratios, fold changes, *p*-values, etc.). The default is that higher values are assigned darker colors. The program automatically adjusts the color distribution based on the current value range. Therefore, users can more easily identify differences between low and high values. In addition, users can adjust the color range to explore entity value distributions over a narrower or wider value range. In addition, entity icons within each compartment are arranged from low value (light colors) to high value (dark colors). This coloring strategy facilitates users being able to notice potentially interesting distributions of entities across particular compartments and categories.

### Automatic scaling

BEV uses the size of each compartment space in the cellular map view and the icon size to calculate how many entity icons the view can accommodate. When loading a file that contains a long list of entities, the number of entities may exceed the maximum number of icons that the area of a compartment in the image can accommodate. In this situation, the program will use one icon to represent multiple entities. The numbers of entities per icon that are displayed is determined by the number of entities that need to be represented and the space available in the image of the cell. In this way, BEV can accept and display inputs with any number of entities. A disc shape is used to represent a single entity. When a unit represents several entities, its shape is displayed as rectangular rather than round. This information is noted in the legend panel.

### Calling up detailed annotations

To view detailed information about an icon, that icon can be clicked, and the icon's name, value, location, and pathway will be displayed as a list in the annotation panel. The annotation information of all the selected icons is shown in the annotation panel. Annotation of entities shown in multiple compartments is not repeated even if multiple occurrences of them in different compartment are selected. The annotation display for an icon disappears when the user unselects the icon by clicking it again. A new annotation list window opens when a list has too many entities to fit in the allotted space, and the number of entities displayed in a new window can be controlled by the user in the legend panel. Additional detailed information about each entity is provided via AtGeneSearch http://metnet.vrac.iastate.edu/MetNet_atGeneSearch.htm when the user right-clicks an icon. This displays all information related to this entity stored in MetNetDB.

### Pathway tree view

**Figure 3:**
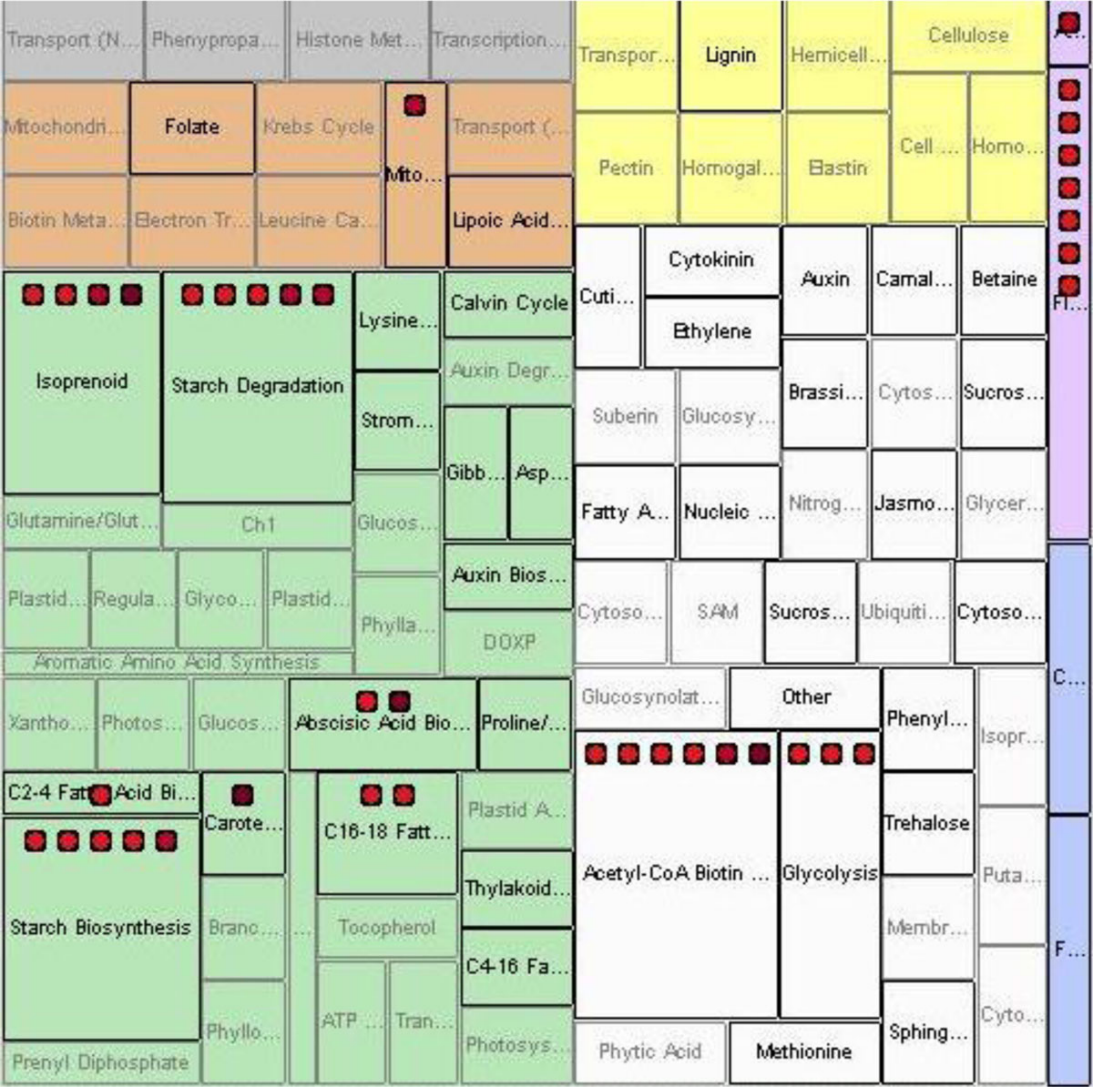
**Pathway Tree map of experimental data within BirdsEyeView**.

The BEV software subdivides the overall Pathway Tree view rectangle. See Figure 3. Within this rectangle, cell compartments provide second-level rectangles. These, in turn, contain third-level rectangles representing the pathways. The Pathway Tree view shows entities in the rectangles of the pathways in which they participate. Pathway rectangles in each cellular compartment rectangle are colored similarly to the Cellular Map view, such that users can see the correspondence between pathways and compartments. Some entities participate in multiple pathways and some pathways are located in multiple compartments; in this case, entities will appear multiple times in the display. As in the Cellular Map view, the arrangement of entity icons into pathway rectangles is based on the pathway information loaded from MetNetDB. Users can perform the same operations on the icons as were described for the Cellular Map view.

The pathways and their corresponding cellular compartments are preloaded by the program from its configuration file and MetNetDB, and are independent of the user input file for the transcriptomic experimental data. Currently, pathways are only known for a small proportion of the known genes, so the Pathway Tree view tends to be the sparsest of the three views. To view the relationships between pathways, cellular compartments, and transcriptomics data, we use the Treemap [32] approach to map biomolecular entities onto different pathways as nested rectangles with entity icons. Treemap is a visualization tool that shows nested rectangles, using the color, size and nesting of rectangles in the graph to reflect hierarchical structure. Each branch of a hierarchy is given a rectangle, which is then tiled with smaller rectangles representing sub-branches. Thus dividing a rectangle into sub-rectangles of specified areas, Treemap can present different trees. The application of Treemap here chooses the pattern of nested rectangles based on the experimental transcriptomic data file and the preloaded pathway information. The more entities a pathway contains, the larger the size of the rectangle representing this pathway. Different transcriptomics data result in different displays of pathway rectangles, enabling users to view pathways in proportion to their size with the transcriptomics data superimposed.

Meanwhile, BEV evaluates overrepresentation of pathways in the data by performing a Fisher exact test for each pathway in which at least one gene from the input list is found. The test is based on the Arabidopsis or the soy genome, depending on which data is input. The Fisher calculation gives the user a list of *p*-values associated with each pathway that indicates how likely it is to for the pathway to contain that number of genes by chance. Each pathway has its *p*-value shown in its mouse-over text. The foreground of each pathway rectangle is highlighted in red if the pathway has a *p*-value satisfying a user-configured threshold for flagging as an over-represented pathway. The threshold can be set in the legend panel. The Pathway Tree view thus provides a simple way for a biologist to gain a better understanding of differentially expressed genes in the context of the pathways they participate in.

### Gene Ontology Biological Process and Molecular Function views

**Figure 4:**
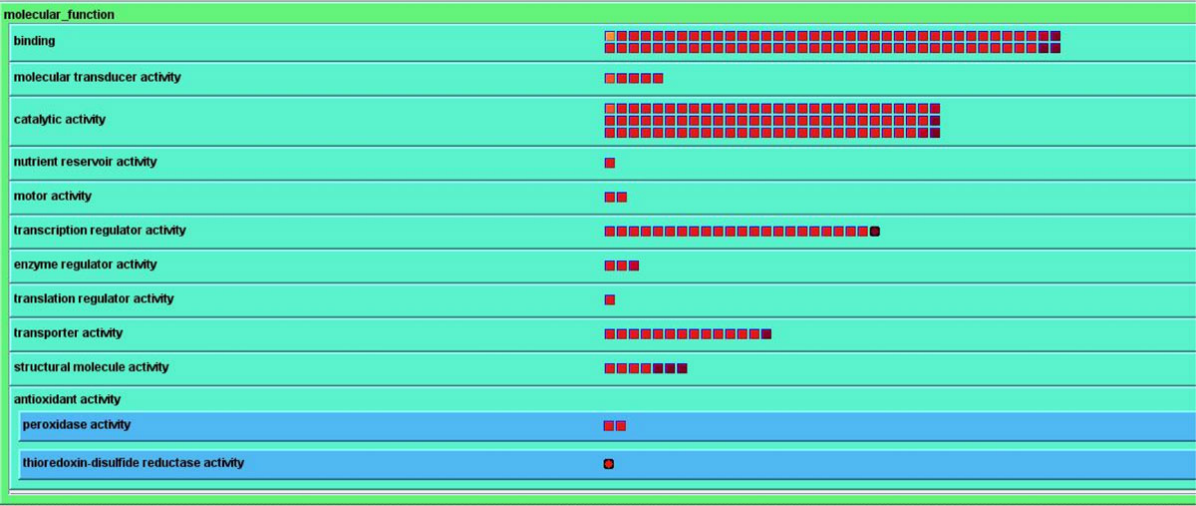
**Gene Ontology view of experimental data within BirdsEyeView**.

BEV provides a Gene Ontology view that displays and maps entities based on current Gene Ontology (http://www.geneontology.org; GO) Biological Process and Molecular Function annotations. GO contains three structured controlled vocabularies (ontologies): biological processes, cellular components and molecular functions. The ontologies are structured as directed acyclic graphs. These can be organized as hierarchies, and one entity may be in multiple locations. The Gene Ontology view shows each GO term (category) as a small rectangle within a series of hierarchically nested rectangles. Experimental entities in each Gene Ontology category rectangle are depicted as small square graphical icons (Figure 4).

BEV first displays all entities as icons in one big rectangle representing the GO root. From the root, users can click the "low" button in the information panel, or click the GO terms of a rectangle hierarchy, to view lower (more specific) level GO categories as smaller sub-rectangles which hold the associated entity icons. This process can be repeated, subdividing rectangles until the lowest (most specific) GO level is reached. By clicking the name of an open category, or clicking the "up" button, users can delete the sub-rectangles in the current rectangle and thus close the current category display and move to a more general category. The color of the rectangles in different levels of the GO hierarchy differ from each other and from entity icons, so that users can easily see the different levels and the icons.

The design of entity icons and access to annotation in the Gene Ontology view is the same as in the Cellular Map view: an icon can represent one or more entities, basic annotation information can be called up in pop-ups with more detail in the annotation panel, and fully detailed annotations can be called up in a new web browser window.

### Data comparisons between samples

BEV supports analysis of experimental transcriptomic data to enable the user to compare data from two samples (e.g. a comparison of gene expression in 3 day old roots vs. 5 day old roots; or control meristems vs. meristems after 10 minutes in 1 M NaCl treatment). For these comparisons across conditions, users can select comparisons of the data by (i) value differences (the difference between the values of the first and second condition); (ii) fold changes (the first condition's values divided by the second condition's values); or (iii) difference folds (the difference between the values of the first and second condition, divided by the second condition's value).

Currently, visualization in BEV is limited to comparison between two groups of data (samples, conditions, etc.); in the future, approaches for overviews of the biological context of multiple samples will be explored. Also, additional statistical analysis can be introduced into BEV to let users extract information from experimental data more conveniently.

### Linked view displays

**Figure 5:**
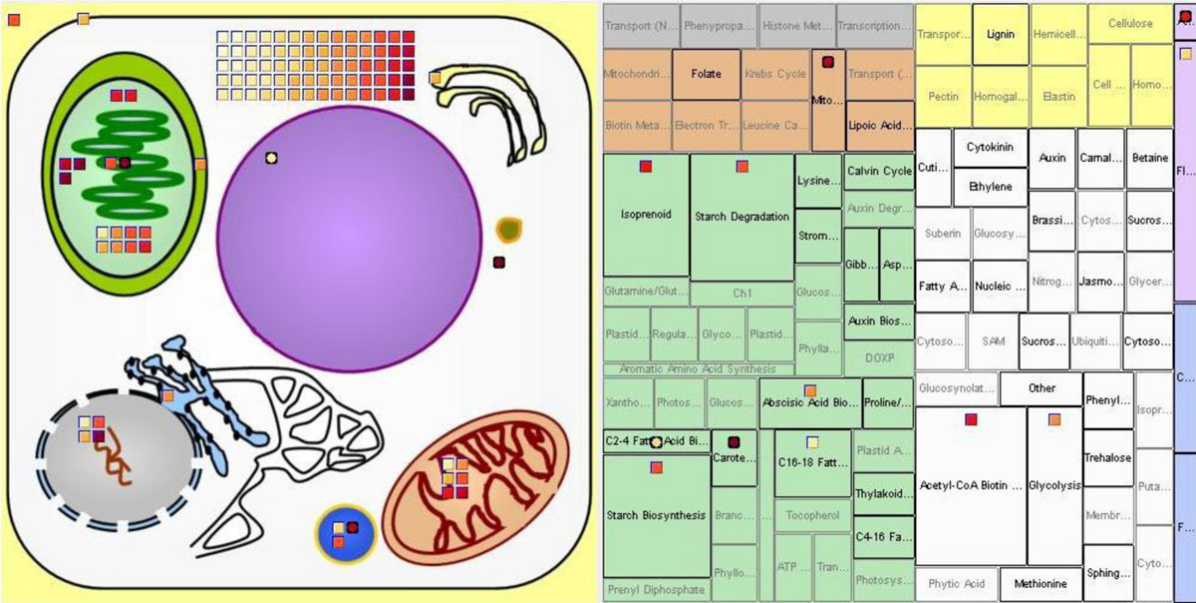
**Combined Cellular Map and Pathway Tree views of experimental data within BirdsEyeView**.

BEV can combine views, allowing users to track entities across different views. Users may choose this option from the view options box. The multiple view option permits all the same operations on the entity icons as does a single view. Because each view in a two-view display has less screen space than on a single view display, fewer details might be visible. For example, in Figure 5 a Cellular Map view and Pathway Tree view are combined, and icons in the Pathway Tree view become single multiple-entity rectangles instead of the larger number of icons that would appear when displaying the Pathway Tree view on its own. By placing views side-by-side, the cellular, pathway, and/or GO properties can be compared simultaneously.

### A case study

**Figure 6:**
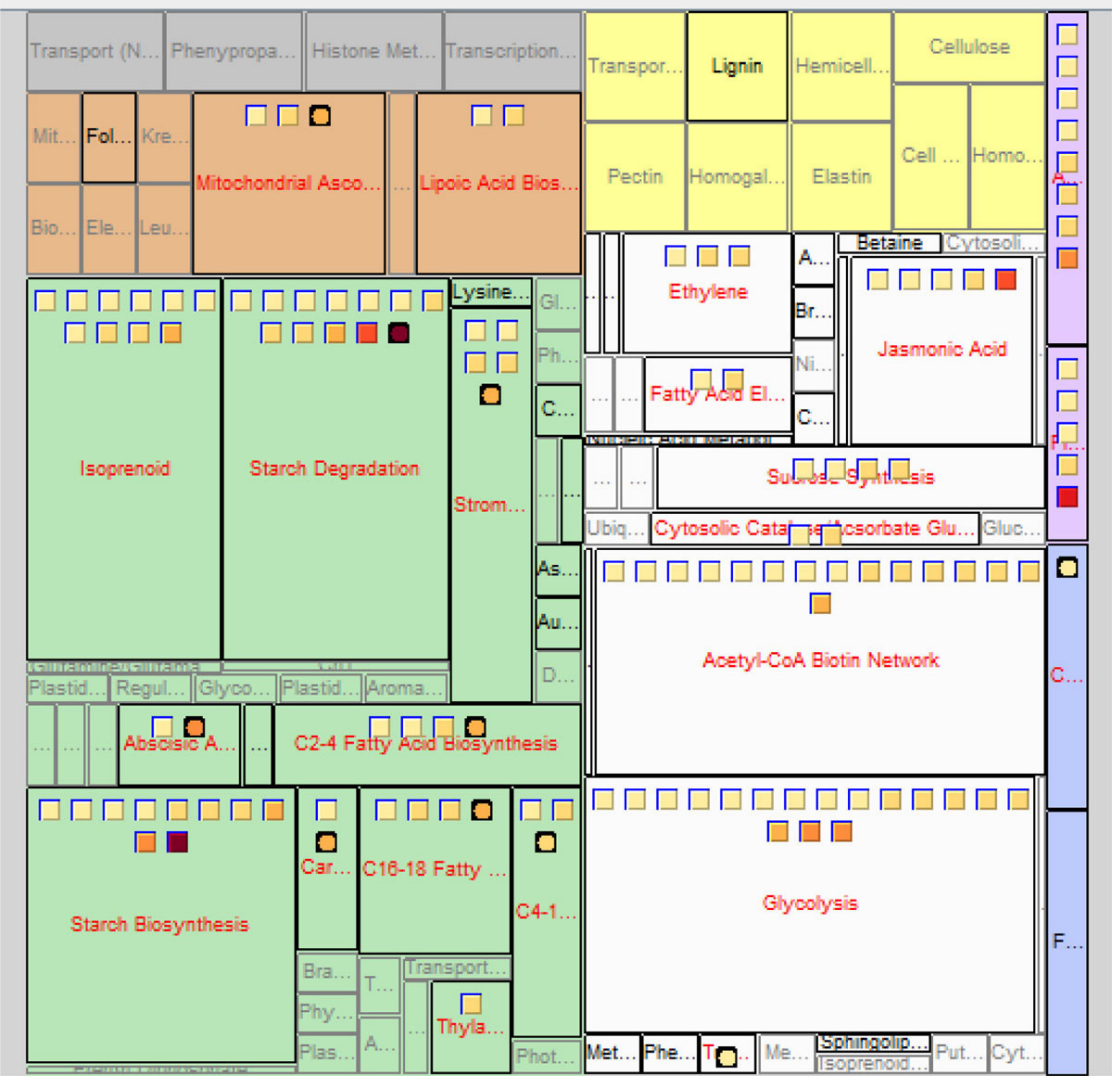
**A case study of soybean seed development: Pathway view of comparison between tissue 'Embryo proper' and 'Endosperm' at 'Globular Stage Embryo'**. Experimental data was obtained from PLEXdb.

Here we use experimental data downloaded from PLEXdb, specifically the means of three replicates for all samples in the experiment "Genomics of Soybean Seed Development Using Laser Capture Microdissection"; this experiment was a microarray-based study designed to investigate the differences in expression levels between different compartments and developmental stages of soybean seeds [33]. Transcripts with data values over 50 in at least one of the samples were uploaded into BEV. Figure 6 shows the Pathway Tree view for experimental data comparison between the samples 'Embryo proper' and 'Endosperm' of seed at the globular embryo stage, using the "fold-change" comparison function in BEV. From Figure 6, it can be seen that several pathways have different numbers of genes with different fold comparison values. For example, the transcripts in 'starch degradation' and 'starch synthesis' have very high fold-increases in the embryo compared to the endosperm, indicating that the pathways related to starch may be more active in the embryo proper than in the endosperm. Starch is the major carbohydrate storage form for chemical energy, and is very important in plant metabolism. We might hypothesize that the gene expression reflects that the embryo is more active in allocating energy than the endosperm during the globular stage of soybean seed development.

## Conclusions

BEV was developed as an alpha version in 2009. The software has been improved and expanded in 2012 based on user input. BEV provides cell, pathway, GO Biological Process, and GO Molecular Function views for experimental transcriptomic data. Users can view their transcriptomics data in the context of biological information, and can flexibly focus in on potentially interesting entities, cell compartments, pathways, biological processes and molecular functions relevant to their experimental conditions. These capabilities help support the needs of systems biologists to view transcriptomics data both in broader contexts as well as in considerable detail. Together with statistical tools such as R, Bioconductor [34], exploRase [35], large data set analysis software such MetaOmGraph [29], and the extant scientific literature, BEV provides a convenient tool to assist in hypothesis development.

## List of abbreviations used

BEV: BirdsEyeView; MetNetDB: MetNet database; GO: Gene Ontology.

## Competing interests

The authors declare that they have no competing interests.

## Authors' contributions

LZ designed the algorithms and developed the BEV software. DB, DC, and ESW determined the goals, system architecture and usability design. YW and LL contributed to interactions between MetNetDB and BEV interactions. All authors contributed to the manuscript.
